# c-di-AMP: An Essential Molecule in the Signaling Pathways that Regulate the Viability and Virulence of Gram-Positive Bacteria

**DOI:** 10.3390/genes8080197

**Published:** 2017-08-07

**Authors:** Tazin Fahmi, Gary C. Port, Kyu Hong Cho

**Affiliations:** 1 Department of Biology, Indiana State University, Terre Haute, IN 47809, USA; tfahmi@sycamores.indstate.edu; 2 Department of Molecular Microbiology, Washington University School of Medicine, Saint Louis, MO 63110, USA; garyport@gmail.com; 3 Elanco Animal Health, Natural Products Fermentation, Eli Lilly and Company, Indianapolis, IN 46285, USA

**Keywords:** c-di-AMP, Gram-positive bacteria, c-di-AMP synthesis and degradation, c-di-AMP-binding proteins

## Abstract

Signal transduction pathways enable organisms to monitor their external environment and adjust gene regulation to appropriately modify their cellular processes. Second messenger nucleotides including cyclic adenosine monophosphate (c-AMP), cyclic guanosine monophosphate (c-GMP), cyclic di-guanosine monophosphate (c-di-GMP), and cyclic di-adenosine monophosphate (c-di-AMP) play key roles in many signal transduction pathways used by prokaryotes and/or eukaryotes. Among the various second messenger nucleotides molecules, c-di-AMP was discovered recently and has since been shown to be involved in cell growth, survival, and regulation of virulence, primarily within Gram-positive bacteria. The cellular level of c-di-AMP is maintained by a family of c-di-AMP synthesizing enzymes, diadenylate cyclases (DACs), and degradation enzymes, phosphodiesterases (PDEs). Genetic manipulation of DACs and PDEs have demonstrated that alteration of c-di-AMP levels impacts both growth and virulence of microorganisms. Unlike other second messenger molecules, c-di-AMP is essential for growth in several bacterial species as many basic cellular functions are regulated by c-di-AMP including cell wall maintenance, potassium ion homeostasis, DNA damage repair, etc. c-di-AMP follows a typical second messenger signaling pathway, beginning with binding to receptor molecules to subsequent regulation of downstream cellular processes. While c-di-AMP binds to specific proteins that regulate pathways in bacterial cells, c-di-AMP also binds to regulatory RNA molecules that control potassium ion channel expression in *Bacillus subtilis*. c-di-AMP signaling also occurs in eukaryotes, as bacterially produced c-di-AMP stimulates host immune responses during infection through binding of innate immune surveillance proteins. Due to its existence in diverse microorganisms, its involvement in crucial cellular activities, and its stimulating activity in host immune responses, c-di-AMP signaling pathway has become an attractive antimicrobial drug target and therefore has been the focus of intensive study in several important pathogens.

## 1. Introduction

Living organisms receive and process environmental stimuli through signal transduction pathways and respond through differential regulation of various cellular processes. Cyclic nucleotides that act as second messenger molecules play key roles in signaling pathways that sense environmental changes such as stress, temperature, nutrition, and pH in both prokaryotes and eukaryotes [1,2,3]. As second messengers, these cyclic nucleotides are involved in the transmission of the signals to effector molecules [1,4]. 

The original second messenger molecule discovered was cyclic adenosine monophosphate (cAMP) through its role in metabolic pathways of eukaryotes, specifically, mammalian hormonal regulation [5,6]. Since its initial discovery, cAMP has been found to act as a second messenger in a variety of organisms including many bacterial species and has diverse activity including roles in biofilm formation, expression of virulence factors, motility, and carbon metabolism [7]. Subsequently, several additional cyclic nucleotide second messengers have been discovered including cyclic guanosine monophosphate (cGMP), cyclic di-guanosine monophosphate (c-di-GMP), cyclic di-adenosine monophosphate (c-di-AMP), and cyclic guanosine monophosphate-adenosine monophosphate (cGAMP) in a wide variety of organisms [2,8,9]. cGMP and c-di-GMP have been well characterized, particularly in Gram-negative bacteria. cGMP is involved in chemotaxis and UV stress-response, while c-di-GMP facilitates the transition from motile phase to adhesive phase and the expression of fimbriae in bacteria [3]. 

c-di-AMP is a new addition to the growing list of second messenger nucleotides and has since been identified in Gram-positive bacteria including *Listeria monocytogenes*, *Bacillus subtilis*, *Staphylococcus aureus*, and *Streptococcus* spp., and in a few Gram-negative bacteria including *Chlamydia trachomatis* and *Borrelia burgdorferi* [1,10,11,12,13,14,15,16]. c-di-AMP has been implicated in diverse essential cellular processes including cell wall and membrane homeostasis, regulation of potassium ion channels, DNA damage repair, and sporulation (Table 1). Though c-di-AMP has been shown to play a critical role in many human pathogenic bacteria, neither its environmental stimuli nor the mechanisms controlling the regulation of cellular physiology and virulence are well understood [15,17]. 

c-di-AMP was first identified in a study of the DNA repair mechanism in *Thermotoga maritima* [29], and later, the same role of c-di-AMP in DNA repair was identified in *B. subtilis* [18,29]. In a separate line of research, c-di-AMP was isolated from the cytosol of *L. monocytogenes*-infected host cells that were producing elevated levels of interferon β [16]. Interferon β is secreted by immune cells in response to infection by microorganisms, and it induces the innate immune response in the host. The correlation between the presence of c-di-AMP and the higher level of interferon β expression indicated that c-di-AMP plays a role in eliciting an immune response in the infected host. Because many human pathogenic Gram-positive bacteria such as *Mycobacterium tuberculosis*, *Streptococcus pyogenes, S. aureus*, and *L. monocytogenes* produce c-di-AMP, the signaling pathway has recently become an attractive drug target [30]. c-di-AMP is essential for the growth of many Gram-positive bacteria such as *S. aureus*, *L. monocytogenes* and *B. subtilis*, so complete depletion of c-di-AMP by deleting the diadenylate cyclase genes in those bacteria leads to the lethal phenotype under standard lab growth conditions unless a special medium is provided, or a suppressor mutation develops [31,32]. On the contrary, the deletion of the diadenylate cyclase gene in *Streptococcus mutans*, *cdaA* was not lethal, suggesting that some bacteria can survive in the absence of c-di-AMP [24]. 

Interestingly, although some bacteria such as *B. subtilis* and *L. monocytogenes* produce both c-di-AMP and c-di-GMP, *S. aureus* and *S. pyogenes* are unable to synthesize c-di-GMP since they lack c-di-GMP synthesizing enzymes [26]. Although most second messenger molecules utilize similar mechanisms in their signaling pathways, their contributions to cell physiology and function differ greatly. Each of the second messenger nucleotides bind to different sets of proteins or RNA molecules, which thereby regulate distinct cellular processes [17]. c-di-AMP works in the signaling pathway in a manner similar to other second messenger molecules such as c-di-GMP, cAMP, and cGMP, but the environmental stimuli and detailed mechanisms are not yet known. 

## 2. Synthesis of c-di-AMP

**Figure 1:**
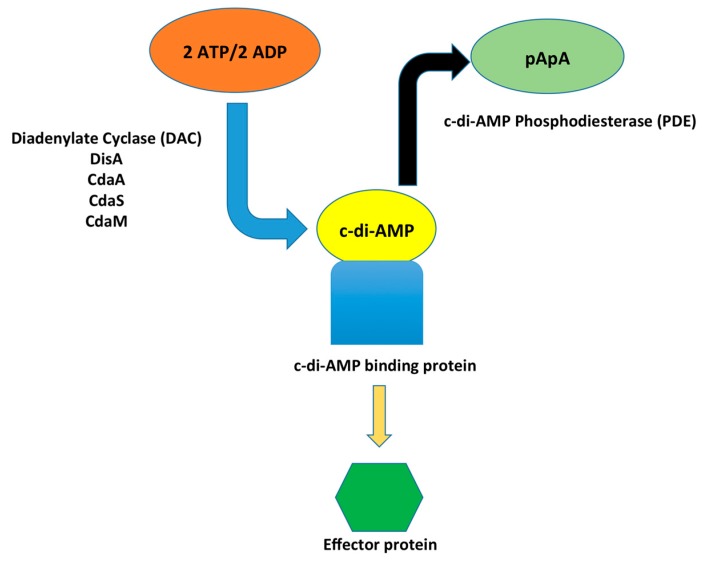
Synthesis and degradation of cyclic di-adenosine monophosphate (c-di-AMP). Diadenylate cyclase (DAC) enzymes synthesize c-di-AMP through a condensation reaction of two ATP or two ADP molecules. c-di-AMP binds to specific target proteins, thereby regulating the functions of downstream proteins within a variety of cellular pathways. To maintain appropriate levels of c-di-AMP, phosphodiesterases (PDEs) degrade c-di-AMP into pApA, which further degrades into AMP [22,43].

In contrast to the variety of GGDEF domain-containing proteins that synthesize c-di-GMP [33], only a few c-di-AMP synthesizing enzymes have thus far been discovered in bacteria and archaea. These c-di-AMP synthesizing enzymes are found primarily in Gram-positive Firmicutes and Actinobacteria but are also present in some Gram-negative bacteria including Bacteroidetes, Deltaproteobacteria, and Cyanobacteria [3]. Moreover, the signaling pathways for c-di-GMP and c-di-AMP rarely co-exist in the same organisms. For example, *Staphylococcus*, *Streptococcus*, and *Corynebacterium* species do not contain functional c-di-GMP synthesizing enzymes [17,34,35]. Notable exceptions to this rule include *Bacillus*, *Clostridium*, *Listeria*, *Mycobacterium*, and *Streptomyces*, which produce both signaling molecules [3,32]. 

c-di-AMP is synthesized from ATP or ADP by cyclase domain-containing proteins known as diadenylate cyclases (DACs) (Figure 1). DAC enzymes catalyze the synthesis of a single molecule of c-di-AMP from two molecules of ATP or ADP through a condensation reaction [3,14,32,34,35]. Structural and functional analysis of individual DAC domains and operons have enhanced the current understanding of the functions of the various classes of these proteins [32,35]. The DAC domain was first identified by X-ray crystallography in DisA (DNA integrity scanning protein A), which functions as a DNA check point protein scanning the chromosome for lesions [18,29]. Later, DAC domain-containing proteins were found in many bacterial and archaeal species [34]. Four classes of DACs have been identified: DisA, CdaA, CdaS, and CdaM. While most organisms contain only one type of DAC, some contain multiple enzymes including *Clostridium* spp., which contain two types of DACs (CdaA and DisA) and *B.subtilis*, which has DisA, CdaA, and CdaS [36]. Several Gram-positive human pathogenic bacteria including *S. pyogenes, S. pneumoniae*, and *L. monocytogenes* encode CdaA [14,16,25] while *M. tuberculosis* expresses MtDisA, a DisA homolog [11,37]. Previous studies have demonstrated that DAC mutant strains display altered physiologies such as loss of resistance to heat, salt, and DNA-damaging molecules due to the synthesis of a weak cell wall, making the bacteria vulnerable to its environment [15,32]. The impact of DAC on numerous aspects of cell physiology highlights the essential nature of DACs in bacteria [15,19,29,32].

All DAC domain proteins possess conserved motifs, most commonly DGA and RHR motifs, which perform the cyclase activity in the c-di-AMP synthesis reactions [38,39]. However, these domains do not share any structural or amino acid similarities with the GGDEF domain found in c-di-GMP-catalyzing enzymes suggesting these signaling pathways evolved independently [40]. Structural analysis of the three types of DACs have demonstrated that the DAC domains in CdaA and CdaS share 40% amino acid identity, while the DAC domain in the DisA protein is more distantly related, sharing only 19% identity with CdaA and CdaS [32,34]. The association of neighboring genes with each class of DAC points to the physiological role of the c-di-AMP. For example, the association of *cdaA* with a gene that encodes an enzyme synthesizing a cell wall building block (*glmM*) suggests a role for CdaA and c-di-AMP in the maintenance of cell wall homeostasis [32,34].

### 2.1. DisA

DisA, crystalized from *Thermotoga maritima*, was the first protein with a DAC domain to be characterized [29]. DisA is found in both Gram-negative and Gram-positive bacteria but is particularly prevalent in Gram-positive spore-forming bacteria such as *Bacillus* and *Clostridium* species. X-ray crystallography revealed that the DAC domains of two tetrameric DisA molecules interact to form a stable octameric structure. The direct contact of DAC domains is necessary for c-di-AMP production [29]. The carboxyl end of DisA enzyme contains a DNA binding domain, and the amino terminal end possesses a globular domain with catalytic activity. These two domains are connected by a helical domain [18]. RadA, a DNA repair protein in *B. subtilis*, is encoded together with DisA in a conserved operon, and their close genetic proximity suggests the involvement of DisA in maintaining DNA integrity [18,32]. Indeed, DisA mutant strains are less able to repair damaged DNA than the wild type [32,34]. It turned out that DisA is a checkpoint protein that directly regulates DNA repair mechanisms by responding to DNA damage [18]. During sporulation in *B. subtilis*, DisA scans DNA and detects damage of chromosomal breaks via its DNA-binding domain, thereby signaling a stop in the sporulation process [17,18]. After repairing the DNA damage, c-di-AMP signals to restart the sporulation pathway [18]. Both high and low c-di-AMP levels impair the DNA binding activity of DisA [18]. 

### 2.2. CdaA

Among the three DACs, CdaA, sometimes referred to as DacA, is the most common as it is found in a wide variety of bacteria including notable human pathogens *S. aureus, S. pneumoniae, S. pyogenes*, and *L. monocytogenes* [17,39]. c-di-AMP produced by CdaA has been shown to be involved in maintaining cell wall homeostasis as well as controlling potassium ion channel activity [32]. The *cdaA* gene is often located immediately upstream of the gene encoding GlmM, a critical peptidoglycan biosynthetic enzyme [32,41]. Bacteria that rely solely upon CdaA for c-di-AMP production develop weakened cell walls, which manifests as a gain in antibiotic resistance when c-di-AMP levels are altered. Furthermore, alterations in c-di-AMP production results in reduced potassium ion channel activity (see KtrA below) [41,42]. In *B. subtilis*, CdaA directly regulates cell wall homeostasis through interaction with GlmM [38]. The CdaA encoding operon also encodes a regulatory protein CdaR, and the interaction of CdaA and CdaR regulates potassium ion channel activity [19,38]. Due to its crucial role in multiple physiological processes, the deletion of the *cdaA* gene is often unsuccessful or only possible under certain circumstances (acquisition of secondary suppressor mutations or specific growth conditions), thus making CdaA an attractive novel target for antibacterial drugs. 

### 2.3. CdaS

The third type of DAC, CdaS, is found only in the spore-forming *Bacillus* species and one *Clostridium* species [32] and is expressed exclusively during spore germination [19]. CdaS-mutant strains have a two-fold decreased germination rate compared with wild-type strains, but the mechanism of CdaS-dependent regulation of sporulation has yet to be revealed. CdaS contains two N-terminal α-helices that are linked to the C-terminal DAC domain [19]. CdaS forms a hexamer that displays relatively low catalytic activity, but a truncated CdaS engineered to lack one or both helices results in a monomer that is hyper-active in c-di-AMP production, indicating that the N-terminal helices are involved in hexamer formation as well as regulation of enzymatic activity. 

### 2.4. CdaM

The fourth class of c-di-AMP synthesizing enzyme, CdaM, has been recently identified in *Mycoplasma pneumoniae* through pull-down assay [28]. CdaM only exists in *M. pneumoniae*, and it is closely related to the DAC domain of CdaS present in *B. subtilis* [28]. *CdaM* mutant strains were unable to grow, indicating that c-di-AMP is essential for the survival of *M. pneumoniae*, [28]. In this bacterium, c-di-AMP also regulates potassium ion transportation. c-di-AMP binds to KtrC, and this interaction interrupts the activity of the low-affinity potassium ion transporter, KtrCD [28]. 

## 3. c-di-AMP Degradation

Bacteria utilize both synthesis and degradation enzymes to regulate the cellular level of second messenger molecules. The c-di-AMP hydrolyzing enzyme phosphodiesterase (PDE) was first identified in *B. subtilis* and was subsequently found in *S. aureus*, *L. monocytogenes*, and *Streptococcus* species [44,45]. PDE enzymes degrade c-di-AMP, converting it into the linear form of phosphoadenyl adenosine (pApA), which can then be further degraded into two molecules of AMP [22,43]. Three classes of PDE are involved in c-di-AMP degradation; GdpP, Pde2 and PgpH [44,45]. The presence of each class of PDE varies by bacterial species. Some bacteria, such as *L. monocytogenes*, encode GdpP and PgpH whereas others, such as *Streptococcus* and *Staphylococcus* species, possess GdpP and Pde2 [44]. The PDE enzymes appear to be stimulated by internal and external stimuli, but the specific stimuli and detailed mechanisms are not yet known. 

### 3.1. GdpP

The best-characterized PDE, GdpP, contains the catalytic DHH/DHHA1 domain (DHH stands for Asp-His-His) [44,45]. This domain is primarily found in phosphatases and phosphodiesterases that regulate the phosphorylation of proteins and breakdown of phosphodiester bonds, respectively [35]. The DHH/DHHA1 domain in GdpP cleaves c-di-AMP into pApA [45]. GdpP is highly specific for c-di-AMP and has been observed to have only very weak enzymatic activity toward c-di-GMP [16,34,45]. GdpP and GdpP homologs have been identified in most microorganisms that produce c-di-AMP thus far [45]. The operon containing the *gdpP* gene appears to be evolutionarily conserved amongst all GdpP producing bacteria as *gdpP* is co-transcribed with the genes of ribosomal protein L9 (RpL9) and a DNA replication protein, DnaC [36,46]. Thus, the co-expression of GdpP, RpL9, and DnaC likely leads to a decrease in the c-di-AMP level during cell growth and replication [36,46]. GdpP also possesses a PAS (Per-Arnt-Sim) domain that is involved in phosphodiesterase inhibiting activity. PAS domains are present in many signaling protein molecules and play critical roles as sensory domains in signal transduction pathways [45]. The PAS domain in GdpP can bind to heme, which inhibits the enzymatic activity of GdpP [47]. GdpP also contains a degenerated c-di-GMP synthesizing domain, GGDEF. However, this domain is not able to synthesize c-di-GMP, but, rather, is involved in the degradation of ATP [45]. A mutation in the GGDEF domain causes reduced catalytic activity of GdpP, thus demonstrating a role of the GGDEF domain in the regulation of GdpP catalytic activity [45].

### 3.2. PgpH

Another class of phosphodiesterase, PgpH, was first identified in *L. monocytogenes*, and now appears to be widespread throughout multiple bacterial phyla [44,48]. PgpH possesses a catalytic histidine-aspartate domain (HD) in its C-terminus that binds to c-di-AMP with high specificity and degrades it into 5’-pApA. HD is composed of two subdomains and two active sites that require iron for their catalytic activity [44]. In addition to an HD domain, PgpH also possesses an N-terminal extracellular domain and seven transmembrane helices [44,48]. In *L. monocytogenes*, PgpH and PdeA, a homolog of GdpP, regulate the intracellular level of c-di-AMP in a cooperative manner. However, these enzymes are regulated by different external stimuli. PgpH expresses preferably in broth culture while PdeA is preferentially expressed during intracellular infection of eukaryotic host cells [44]. *L. monocytogenes* mutants lacking either PgpH or PdeA exhibit slight growth defects compared to the wild type. However, mutants lacking both *pdeA* and *pgpH* exhibit higher levels of c-di-AMP, which is detrimental to bacterial growth and contributes to reduced virulence of *L. monocytogenes* in a mouse model of infection, indicating the cooperative c-di-AMP degradative activity of these two enzymes [44]. The double mutant also elicits increased interferon (IFN) - β during intracellular infection of host cells [48]. Upon infection, *L. monocytogenes* induces the expression of β -interferon and co-regulated genes following stimulation of the cytosolic surveillance pathway (CSP), STING, and DDX41 pathways in the host immune system [21]. In a screen designed to determine the Pathogen Associated Molecular Patterns (PAMPs) recognized by the infected mammalian cells, c-di-AMP was identified [16]. Indeed, bacterial mutants that secrete more c-di-AMP lead to an increase in β-interferon secretion [21]. 

### 3.3. Pde2

Pde2 is a recently discovered c-di-AMP degrading enzyme containing a DHH/DHHA1 domain. It was first identified in *S. pneumoniae* [43,49] and its enzymatic activity and structures have been studied in *S. aureus* [50], *Mycobacterium* spp. [51,52,53], and *Borrelia burgdoferi* [54]. *M. pneumoniae*, *B. burgdorferi*, and *M. tuberculosis* contain only PDEs of the Pde2 type. Pde2 is a cytoplasmic protein that can degrade c-di-AMP but preferentially hydrolyzes linear pApA to AMP as demonstrated in *S. aureus* [43,50]. Deletion of the *pde2* gene leads to a rapid increase of intracellular pApA levels compared to the wild type. pApA and c-di-AMP concentrations are interconnected as pApA inhibits the c-di-AMP hydrolyzing activity of GdpP [50]. Thus, Pde2 plays a key role in maintaining the homeostasis of intracellular pApA and c-di-AMP levels that are crucial for the growth and survival of bacteria.

## 4. c-di-AMP Binding Molecules

Since the diverse roles of c-di-AMP in cell signaling pathways depend upon its binding to target molecules, the investigation into the identification, structure, and function of c-di-AMP binding molecules is important to reveal the mechanisms of c-di-AMP activity in various signaling pathways. However, unlike c-di-GMP for which hundreds of binding proteins have been identified [32,33,55], a few c-di-AMP binding proteins have been discovered so far in bacteria (Table 2) [17]. Multiple strategies have been successfully employed to identify c-di-AMP binding proteins including UV-crosslinking of ^32^P-labelled c-di-AMP with a library of purified proteins [55]. However, the most common strategy involves the use of c-di-AMP affinity columns to isolate binding proteins from bacterial cytoplasmic extracts followed by gel electrophoresis and mass-spectroscopy [17,25,48]. c-di-AMP binding is typically confirmed via standard affinity assays such as surface plasmon resonance (SPR) or pulse chase analysis [55], or more recently through an adaption of the differential radial capillary action of ligand assay (DRaCALA) first used to study c-di-GMP binding [17,48]. These recently identified c-di-AMP binding proteins include enzymes, transporters and transcriptional regulators that are themselves allosterically regulated by c-di-AMP binding. The structural analysis of c-di-AMP binding proteins has also facilitated the identification of conserved c-di-AMP binding-domains, thus leading to the discovery of additional c-di-AMP binding proteins. In addition to binding proteins, c-di-AMP can bind to RNA riboswitches [56,57,58,59,60].

### 4.1. TetR-Family Transcription Factor, DarR

DarR from *Mycobacterium smegmatis*, a member of the TetR-family regulator proteins, was identified through a c-di-AMP transcription factor binding screen [38,55]. TetR regulators are DNA binding proteins whose affinity towards DNA is altered upon interaction with their cognate small molecule. Following small molecule interaction, these regulators undergo a structural change, thereby altering their affinity towards specific DNA binding sites and giving rise to the altered expression of target genes. Like the other members of the TetR family, DarR represses its own promoter as well as the downstream genes. DarR homologs are found primarily in Gram-positive bacteria such as *Bacillus* spp., *Staphylococcus* spp., *Listeria* spp., *Lactobacillus* spp., and *Clostridium* spp. [38,55].

DarR contains two domains: a C-terminal QacR-like domain, and an N-terminal TetR-like helix-turn-helix DNA binding domain [38,55]. Interestingly, not all c-di-AMP-producing bacteria possess QacR-like domains in their DarR proteins. The domain is found in *Staphylococcus*, *B. subtilis*, and *Pelotomaculum thermopropionicum* [55]. The detailed mechanism of c-di-AMP allosteric regulation of DarR is not well understood, nor has the binding site of c-di-AMP been identified, but c-di-AMP has been shown to stimulate the binding of DarR to its target DNA [55]. DarR binds to the promoter regions of genes that are involved in fatty acid metabolism and the expression of the cold shock protein in *M. smegmatis* as *darR* deleted *M. smegmatis* exhibits larger cell size and reduced fatty acid metabolism [38,55]. 

### 4.2. RCK_C Domain Protein KtrA

The first c-di-AMP binding protein isolated and characterized from bacterial cytoplasmic extract through c-di-AMP affinity chromatography was potassium transport protein, KtrA, an RCK (regulator of conductance of K^+^) domain-containing protein from *S. aureus* [17]. RCK domains are commonly found in potassium ion transport systems and are involved in the regulation of ion channel gating [58]. KtrA possesses two RCK domains, an N-terminal RCK (RCK_N) domain that is able to bind to a variety of molecules such as ATP, ADP, NAD, and NADH, and a C-terminal RCK (RCK_C) domain that binds to c-di-AMP [58]. KtrA associates with KtrB, a membrane-integrated protein that forms the potassium ion channel [65]. c-di-AMP binding to KtrA inhibits potassium transport [17,20,25] Furthermore, *ktrA* mutated *S. aureus* exhibits lower survival rates than the wild type strains when bacteria are grown in medium containing low potassium concentrations [17,32]. The link between c-di-AMP and potassium ion channel proteins is found not only in *Staphylococcus* strains but also in *Bacillus* and *Streptococcus* species [17,20,25].

### 4.3. RCK_C Domain Protein CpaA

Another c-di-AMP binding protein isolated from *S. aureus* is cation-proton antiporter, CpaA [17]. CpaA was identified bioinformatically based on the presence of the RCK domain, as found in KtrA, but structurally, these two c-di-AMP binding proteins have very low overall identity to each other. CpaA was confirmed to bind labeled c-di-AMP by DRaCALA assay. CpaA is a predicted cation/proton anti-porter that exchanges intracellular protons with potassium, but may also transport sodium and calcium ions [17]. A recent structural study revealed that c-di-AMP binding alters transport activity by 40% [66]. 

### 4.4. Histidine Kinase Protein, KdpD

KdpD is yet another c-di-AMP binding protein identified from *S. aureus*, this time by screening a recombinant *S. aureus* ORFeome expression library using DRaCALA [17]. KdpD is a histidine kinase that functions as a two-component system (TCS) with KdpE, the response regulator. Generally, TCSs are composed of a histidine kinase and a cognate response regulator. The histidine kinase spans the cell membrane and senses intracellular or extracellular signals including changes in pH, osmolarity, or quorum sensing molecules. Upon sensing appropriate stimuli, the histidine kinase transduces this information via phosphotransfer to a cytosolic response regulator protein that generally functions as a transcriptional regulator. KdpD has been shown to regulate potassium homeostasis in the cell by altering the expression of genes involved in the Kdp high-affinity potassium uptake system [62]. This potassium uptake system is ATP-dependent and responds to very low potassium conditions where other systems cannot support potassium uptake [32]. KdpD-mutated *S. aureus* displays dysregulated ion channel systems, which lead to lower virulence and decreased survival of bacteria as compared to the wild type. c-di-AMP binds to the universal stress protein domain (USP) within the N-terminal cytoplasmic region of KdpD and inhibits the upregulation of the genes in the Kdp potassium uptake system under salt stress [67]. 

### 4.5. PII-Like Signal Transduction Protein, PstA

Another c-di-AMP binding protein discovered using the ORFeome DRaCALA screening technique is PstA, first identified in *S. aureus* but also present in *L. monocytogenes* and *B. subtilis* [32,35]. PstA is a predicted cytoplasmic PII-like signal transduction protein. Among the members of the PII-like signal transduction proteins, the PII nitrogen regulatory protein is the best characterized. This protein detects the amount of nitrogen and carbon in cells by sensing glutamine and 2-oxoglutarate levels [64]. The analyses of the crystal structures of PstA in three microorganisms, *S. aureus*, *L. monocytogenes* and *B. subtilis* revealed that PtsA forms a homotrimer and c-di-AMP binds a pocket located between two subunits, so three molecules of c-di-AMP bind to the homotrimer [64,68,69]. 

### 4.6. KtrA Homolog Protein, CabP

CabP was identified in *S. pneumoniae* by c-di-AMP affinity chromatography of bacterial cell lysates [25]. This protein belongs to the potassium ion transporter protein family, Trk , which is involved in potassium ion transport. CabP has a molecular mass of 24 kDa and is 33% identical to the KtrA protein by amino acid sequence, both of which form stable octamers in solution [17,25]. The presence of genes encoding CabP and the KtrB ortholog, SPD_0076, in the same operon in *S. pneumoniae* further supports the involvement of CabP in potassium uptake [25]. Indeed, deficiency of either CabP or SPD_0076 leads to impaired potassium uptake and decreased survival of the bacterium [25]. Using an *E. coli* two-hybrid system coupled with coexpression of the c-di-AMP synthesis enzyme DisA (as *E. coli* does not normally produce c-di-AMP), c-di-AMP was shown to inhibit the interaction of CabP with SPD_0076 and thereby lead to the reduction of potassium uptake [25]. CabP has a high affinity for c-di-AMP, and the presence of other nucleotides such as cAMP, c-di-GMP, AMP, and pApA does not decrease the CabP:c-di-AMP binding rate. In the above-mentioned study, Bai et al. isolated several proteins from the crude cytoplasmic extract of *S. pneumoniae* and CabP was the most abundant. This suggests that more c-di-AMP binding proteins still await discovery. 

### 4.7. CabPA and CabPB

Two c-di-AMP binding proteins, CabPA and CabPB have been recently identified in *S. mutans* through an affinity chromatography pulldown assay [23]. They belong to the TrkA protein family like KtrA. TrkA protein family members possess a TrkA_N and a TrkA_C domain, and the TrkA_C domain has been shown to be a c-di-AMP binding domain. TrkA family proteins are typically involved in potassium ion transport, but the role of CabPA and CabPB in potassium ion transportation has not been demonstrated [23]. Peng et al. have uncovered a role of c-di-AMP in the regulation of biofilm formation through a comparative study of wild type and PdeA-mutated *S. mutans* strains. Deletion of *pdeA* leads to an increase in the intracellular level of c-di-AMP, thereby leading to decreased cell growth but increased biofilm formation [23]. The *cabPA* deletion suppresses the increased biofilm formation by the *pdeA* deletion [23]. CabPA also interacts with VicR, which is known to regulate the expression of biofilm matrix protein, GtfB. The interaction among CabPA, VicR and GtfB appears to be at the center of the biofilm regulatory network controlled by c-di-AMP [23]. 

### 4.8. LmPC 

c-di-AMP affinity chromatography followed by confirmation with DRaCALA was used to identify several hypothetical proteins from *L. monocytogenes* as well as pyruvate carboxylase renamed LmPC [48]. LmPC is an enzyme used in central carbon metabolism, converting pyruvate to oxaloacetate. Binding studies revealed a specificity of LmPC for c-di-AMP binding compared with structurally related di-nucleotides [48]. Furthermore, binding of c-di-AMP to LmPC decreases catalytic activity of the enzyme, and the LmPC:c-di-AMP interaction therefore regulates various metabolic pathways in *L. monocytogenes* including carbon flux through the citric acid cycle as well as glutamate biosynthesis, processes that are crucial for bacterial survival within the host environment [48]. LmPC is a tetrameric protein that contains several domains including biotin carboxylase (BC) and carboxyltransferase (CT) domains. The tetrameric structure of LmPC is composed of two layers of monomers through the strong interactions of BC and CT domains [48]. Structural analysis revealed that two molecules of c-di-AMP can bind to the dimer interfaces of two CT domains in a two-fold symmetry in a region of the protein that was not previously recognized to have any regulatory significance [48]. Mutations within the c-di-AMP binding site that prevent the LmPC:c-di-AMP interaction alter the catalytic activity of the carboxylase enzyme domain [48]. 

### 4.9. c-di-AMP Binding Riboswitches

Second messenger molecules can bind not only to specific proteins but also to regulatory RNA molecules known as riboswitches [35,56,58]. Many classes of riboswitches have been identified bioinformatically based on the presence of conserved RNA motifs within intergenic regions, and have subsequently been shown to bind a variety of molecules including metals, amino acids, carbohydrates and other second messenger molecules in the cells, and thereby regulate downstream genes by pre- or post-transcriptional mechanisms [59]. Riboswitches generally contain a ligand-sensing domain and an expression platform to facilitate the signal transduction of ligand binding and differential expression of the regulated genes [56]. Although several riboswitch classes have been identified based on the presence of RNA motifs, many of them are still considered “orphan” as no ligand as yet been identified [70]. 

One well-known riboswitch, the *ydaO* riboswitch, since one of them exists in the promoter region of the *B. subtilis ydaO* gene, was found to be associated with genes that regulate cell wall metabolism, osmotic stress, and sporulation. However, the riboswitch ligand remained elusive for many years until an unbiased search for the *ydaO* riboswitch binding molecules utilizing yeast extract as a source of chemically diverse natural metabolites identified AMP as a ligand with weak affinity, and further refinement led to the identification of c-di-AMP as the primary ligand [71,72]. A mutagenic study of *B. subtilis* revealed that the *ydaO* riboswitch is not essential for the bacteria and functions as a transcription terminator in the c-di-AMP bound state. During times of increased c-di-AMP concentration, the riboswitch can act as a genetic off switch to turn off gene expression. The ligand-binding domain of the *ydaO* riboswitch possesses highly conserved residues for c-di-AMP recognition. It also exhibits a novel platform in the c-di-AMP bound state that includes pseudo-two-fold symmetry of the RNA and a very stable conformation formed after binding to two c-di-AMP molecules [57,59]. The binding of two c-di-AMP molecules provides a very stable riboswitch conformation, which supports the idea that two c-di-AMP molecules work cooperatively. This conformation reveals that the binding pattern of c-di-AMP to a riboswitch is very different from the binding of c-di-AMP to proteins. 

The *ydaO* riboswitch is currently the only known c-di-AMP binding riboswitch. Although the riboswitch motif is widely distributed in many bacteria, *ydaO* is present only in some bacteria that produce c-di-AMP, namely *Bacillus* and *Clostridium* species. The *ydaO* riboswitch has not been found in *Lactococcus*, *Listeria*, *Staphylococcus*, or *Streptococcus* [35]. Interestingly, in *B. subtilis*, the *ydaO* riboswitch is located within the *ktrAB* transcript, which encodes a potassium ion transporter (see above), and the *kimA* (aka ydaO) transcript, which encodes a high affinity potassium ion transporter [73]. The c-di-AMP -riboswitch interaction negatively controls the expression of *kimA*. High levels of extracellular K+ increases the intracellular c-di-AMP concentration, which, in turn, represses the expression of riboswitch controlled genes in the potassium transport system [73]. 

## 5. Conclusions

Second messenger molecules are critical elements in signal transduction pathways utilized by many organisms. They are involved in the regulation of various key cellular processes in both prokaryotes and eukaryotes. In recent years, c-di-AMP signaling has been identified as a central factor in many Gram-positive bacteria regulating cell wall synthesis, potassium ion channels, DNA repair, and biofilm formation [32]. c-di-AMP is also essential for cell growth, survival, and virulence of several well-known human pathogenic bacteria including *S. aureus*, *L. monocytogenes*, *S. pyogenes*, and *Mycobacterium* spp. [17,20,22,26,35]. Although many cellular functions of c-di-AMP are known, the detailed mechanisms of the cellular processes have yet to be revealed. The c-di-AMP synthesizing enzymes and degradation enzymes regulate the homeostasis of intracellular c-di-AMP levels. Four classes of DAC (DisA, CdaA, CdaS, and CdaM) and three types of PDE (Gdpp, Pde2 and PgpH) are found in c-di-AMP-producing bacteria, and the types of enzymes and their functions vary by bacterial species [1,32]. Due to their essential role in bacteria, the construction of DAC mutant strains is often difficult. For example, the construction of DAC deletion strains in *L. monocytogenes* was not possible in rich media unless accompanied by a secondary mutation in guanosine pentaphosphate (ppGpp) synthases. Similarly, simultaneous depletion of all three DACs in *B. subtilis* was synthetic-lethal [36]. However, deletion of the DAC enzyme in *S. mutans* was not lethal, so c-di-AMP might be not essential for the growth of some bacteria even under standard lab growth conditions. 

Unlike the c-di-GMP network, where more than twenty enzymes are involved in the production and degradation of the cyclic di-nucleotide, the c-di-AMP synthesis and hydrolysis network appears much less complex. DACs and PDEs regulate intracellular c-di-AMP homeostasis, while the receptor proteins and RNA are the key factors that carry out the cellular processes upon binding to c-di-AMP [32,35]. As DAC is often an essential enzyme in many bacteria, many roles of c-di-AMP in cellular processes have been revealed by examining PDE mutant strains. The alteration of c-di-AMP levels following PDE depletion often results in decreased survival of the bacteria. Furthermore, altered cell wall synthesis and dysregulation of potassium uptake have been observed in PDE mutant strains [17]. 

To understand the regulatory network of c-di-AMP at the cellular level, further intensive molecular studies are necessary. Research has been directed towards the characterization of the receptor molecules to decipher the regulation mechanisms by c-di-AMP. Several proteins and one type of RNA molecule have so far been identified that bind to c-di-AMP (Table 2) [32,35]. It appears that there is not much overlap between c-di-AMP binding proteins among bacteria except the proteins involved in potassium ion homeostasis. Thus, identifying c-di-AMP-binding proteins using the bacteria previously not studied might discover new types of c-di-AMP-binding proteins, resulting in discovering new cellular pathways controlled by c-di-AMP. The only c-di-AMP-binding RNA identified so far is the *ydaO* riboswitch, and many bacteria producing c-di-AMP do not contain this riboswitch. New types of c-di-AMP-binding RNAs might also be discovered by the screening of c-di-AMP-binding RNA in those bacteria. Thus, investigations with diverse organisms might lead to a better understanding of the mechanisms of regulation in c-di-AMP signaling pathways. The specific environmental stimuli in c-di-AMP signaling pathways are still a mystery, so identifying the signals is also necessary for better understanding of how bacteria sense environmental stimuli and adapt to environmental changes. Agents that disrupt c-di-AMP signaling, such as inhibitors of DACs and PDEs, may also prove useful for antibacterial therapy in the future. 

## Figures and Tables

**Table 1:** The function of cyclic di-adenosine monophosphate (c-di-AMP) and its synthesis and degradation enzymes in bacteria.

Bacterium	Function of c-di-AMP	c-di-AMP Synthesis Enzyme	c-di-AMP Degrading Enzyme	Phenotype Involved in an Altered Level of c-di-AMP	Ref.
*Bacillus subtilis*	DisA binds to DNA and maintains DNA integrity. CdaS regulates sporulation. CdaA regulates cell wall synthesis and ion channel homeostasis.	DisA, CdaA, and CdaS	GdpP and PgpH	DisA mutation: decreased DNA integrity CdaA mutation: impaired potassium ion channel system, weakened cell wall, increased resistance to antibiotics CdaS mutation: delayed sporulation.	[17,18,19,20]
*Listeria monocytogenes*	Regulates cell wall homeostasis, resistance to acid, and carbon metabolism.	CdaA (DacA)	PdeA (GdpP homolog) and PgpH	Phosphodiesterase (PDE mutation: cell wall defects, increased resistance to antibiotics, low survival rate, sensitivity towards acid stress, altered interferon-ß stimulation in host cells.	[21]
*Mycobacterium tuberculosis*	Functions are not fully understood yet, but DisA is predicted to be involved in DNA repair.	MtbDisA (DisA ortholog)	MtbPDE (Pde2 ortholog)	PDE mutation: reduced virulence.	[22]
*Staphylococcus aureus*	Regulates cell wall synthesis, cell size, and potassium ion channel homeostasis.	CdaA	GdpP and Pde2 ortholog	*gdpP* deletion: smaller cell size, increased peptidoglycan cross-linking, increased resistance against cell wall and membrane targeting antibiotics, impaired potassium ion channel system.	[17]
*Streptococcus mutans*	Regulates biofilm formation by binding to receptor proteins.	CdaA	PdeA (GdpP ortholog) and Pde2	*cda* deletion: Increased sensitivity to hydrogen peroxide and enhanced polysaccharide synthesis. *pdeA* deletion: Increased biofilm formation.	[23,24]
*Streptococcus pneumoniae*	Maintains potassium ion channel homeostasis.	CdaA	GdpP and Pde2	PDE mutation: Impaired ability of long chain formation, decreased growth, and imbalance in the potassium ion channel.	[25]
*Streptococcus pyogenes*	Regulates cell wall homeostasis and virulence gene expression.	CdaA (SpyDacA)	GdpP and Pde2 ortholog	*gdpP* deletion: Impaired biogenesis of SpeB, decreased virulence and increased antibiotic resistance.	[26]
*Streptococcus suis* (SS2)	Promotes biofilm formation and increases virulence.	CdaA	GdpP and Pde2 ortholog	*gdpP* deletion: Reduced growth and reduced biofilm formation.	[27]
*Mycoplasma pneumoniae*	Predicted to regulate potassium import through binding of KtrC.	CdaM	PdeM	*cdaM* and *pdeM*: essential for growth.	[28]

**Table 2:** c-di-AMP binding molecules.

c-di-AMP Receptor Proteins (Species Originally Identified in)	Location in the Cell	Protein Structure and Functional Domains	Protein Function	Phenotypes by Deletion or Overexpression of the Genes	References
DarR *(Mycobacterium smegmatis,)*	Cytoplasmic protein	DarR contains two domains: a C-terminal QacR-like domain, and an N-terminal TetR-like helix-turn-helix domain. The binding site of c-di-AMP has not been identified yet.	Transcriptional repressor for genes involved in ion transport, membrane lipid homeostasis, and stress response.	Deletion causes larger cell size. Overexpression is toxic to cells and causes reduced fatty acid metabolism.	[38,55]
KtrA *(S. aureus)*	Cytoplasmic protein bound to the integral membrane protein KtrB	KtrA possesses two RCK domains, RCK_N and RCK_C. c-di-AMP binds to RCK_C.	KtrA-KtrB complex regulates the potassium ion channel opening and closing by changing their conformation following c-di-AMP binding.	Deletion causes sensitivity to osmotic stress and requires high levels of potassium for growth.	[17,32,58,61]
CpaA (*S. aureus)*	Integral membrane protein	Twelve membrane-spanning region, RCK_N and RCK_C domain c-di-AMP binds to RCK_C.	A putative proton antiporter in the cell where intracellular protons are exchanged with potassium or sodium ions.	Not studied yet.	[17,32]
KdpD *(S. aureus)*	Integral membrane protein	KdpD is a histidine kinase in a TCS.	Regulates a P-type ATP- dependent high-affinity potassium uptake system.	Deletion leads to low virulence and less survival.	[32,35,62,63]
PstA *(S. aureus)*	Cytoplasmic protein	PII-like signal transduction protein with an unknown domain DUF970.	Unknown.	Not studied yet.	[64]
CabP *(S. pneumonaie)*	Cytoplasmic protein	An octameric protein belonging to the Trk family Homolog of KtrA. Binds to the ortholog of KtrB SPD_0076.	A member of the potassium ion transporter.	CabP mutant exhibits low potassium ion uptake.	[25]
CabPA *(S. mutans)*	Cytoplasmic protein	Trk family.protein.	Binds to VicR, facilitates biofilm formation.	Reduces biofilm formation ability.	[23]
CabPB *(S. mutans)*	Cytoplasmic protein	Trk family protein.	Unknown.	CabPB mutant strains have not been studied yet.	[23]
LmPC (*L. monocytogenes*)	Cytoplasmic protein	Pyruvate carboxylase family, c-di-AMP binds to the dimer interface.	Pyruvate carboxylase: ATP-dependent carboxylation of pyruvate to oxaloacetate.	Causes metabolic imbalance, lysis of bacterial cells during infection.	[48]
CbpA, CbpB (*L. monocytogenes*)	Soluble proteins	Function unknown.	Unknown.	Not studied yet.	[48]
NrdR (*L. monocytogenes*)	Cytoplasmic protein	Transcriptional repressor.	Transcriptional regulator.	Not studied yet.	[48]
*ydaO* Riboswitches *(B. subtilis*)	Cytoplasmic RNA molecules	The regulatory RNA molecules contain a ligand-sensing domain and an expression platform. c-di-AMP binds to the ligand-sensing domain.	Regulates ion channels, responds to osmotic stress, and facilitates cell wall metabolism and sporulation.	Not essential.	[32,35,45,56,57,58,59,60]
